# ‘In my life antidepressants have been…’: a qualitative analysis of users’ diverse experiences with antidepressants

**DOI:** 10.1186/s12888-016-0844-3

**Published:** 2016-05-11

**Authors:** Kerry Gibson, Claire Cartwright, John Read

**Affiliations:** School of Psychology, University of Auckland, Private Bag 92019, Auckland, 1142 New Zealand; School of Psychology, University of East London, London, United Kingdom

**Keywords:** Depression, Antidepressants, Patient experiences, Medication use

## Abstract

**Background:**

While mental health professionals have focused on concerns about whether antidepressants work on a neurochemical level it is important to understand the meaning this medication holds in the lives of people who use it. This study explores diversity in the experience of antidepressant users.

**Methods:**

One thousand seven hundred forty-seven New Zealand antidepressant users responded to an open-ended question about their experience of antidepressants. This was analysed using content and thematic analysis.

**Results:**

There was considerable diversity in participants’ responses including positive (54 %), negative (16 %) and mixed (28 %) experiences with antidepressants. Those with positive experiences saw antidepressants as a necessary treatment for a ‘disease’, a life saver, a way of meeting social obligations, dealing with difficult circumstances or a stepping stone to further help. Negative themes described antidepressants as being ineffective, having unbearable side effects, undermining emotional authenticity, masking real problems and reducing the experience of control. Mixed experience themes showed how participants weighed up the unpleasant side effects against the benefits, felt calmer but less like themselves, struggled to find the one or dosage and felt stuck with continuing on antidepressants when they wished to stop.

**Conclusions:**

Mental health professions need to recognize that antidepressants are not a ‘one size fits all’ solution.

## Background

Antidepressants are being prescribed at high levels across the developed world [1, 2]. In New Zealand, where this study was undertaken, an estimated one in nine adults receives prescriptions every year [3]. Professional debate has typically focused on whether antidepressants ‘work’ on a neurochemical level [4, 5] with users being viewed as relatively passive recipients of medical decision-making [6]. However there is an important body of research recognising that people are actively involved in making meaning of their medication use and that this has implications this has for the value it has in their lives [7–10]. Small scale qualitative research has often highlighted negative experiences [11–14] or noted considerable ambivalence amongst some antidepressant users [15, 16]. Only a very limited amount of research has attempted to understand positive experiences of antidepressants, also using small samples [17].

Research suggests a range of factors predict different experiences of antidepressants, including demographic factors, psychosocial factors, people’s belief about depression, and their relationship with their prescriber [18]. Some users react differently to the same antidepressant [19] while different antidepressants produce different effects [20]. In addition, there is likely to be some fluidity in people’s views through their ‘journey’ with antidepressants [21, 22]. Most of the qualitative studies that inform this area also recognize that while people actively make meaning of their experiences of medication use, they do so in the context of prevailing social ideas that help to shape the way that these can be thought about [23]. Currently people make sense of their antidepressant use against a contested terrain in which antidepressants are represented as an effective strategy for treating a bio-medicalised conception of depression [24] alongside concerns over the over-prescription and misuse of this medication [1, 25].

An understanding of users’ own experiences of antidepressant use is increasingly important in the context of growing doubts about the effectiveness of antidepressant treatment [5, 26], concerns about side effects [27] and withdrawal effects [28]. With less certainty amongst professionals that antidepressants necessarily ‘do some good’ [29], it important to gather feedback on how users themselves experience the value or otherwise of these medications in their lives.

This article analyses the responses of a large sample of antidepressant users to an open-ended survey question on the impact that antidepressants had on participants’ lives. Our analysis aims to explore the potential diversity of experiences with antidepressants and the meanings attributed to them.

## Methods

This article draws from responses to one open question in an anonymous online survey exploring the experience of adult New Zealanders prescribed antidepressants. The question asked participants to complete the phrase: “In my life antidepressants have been…” The study was approved by the University of Auckland, Human Participants Ethics Committee and participants gave informed consent for their participation.

### Participants

Participants were informed of an online survey via widespread media advertising. The criteria for participation included having been prescribed antidepressants in the last five years, living in New Zealand and being 18 years of age or over. The survey was available online from March 2012 until January 2013. There were 1,829 completed surveys for analysis. The majority of the sample was women (77 %). The modal age group was 36-45 (24.2 %); 16.3 % were 18 to 25, and 15.9 % were 56 or older. Population figures suggest that 13.7 % of the New Zealand population is 65 or older [30] while only 3.6 % of our sample were over 65. The sample also appeared to have a somewhat higher education than the general population with 49.6 % reporting they had a university degree; 26.1 % gained a diploma or certificate after high school, 17.2 % completed high school, and 7.1 % did not complete high school as compared with 14.2 % of adult New Zealanders who had an undergraduate degree or higher and 22.4 percent who had no formal qualification in 2006 [31]. The annual income of this sample ranged from less than NZ$10,000 (15.0 %) to over NZ$100,000 (7.7 %). The modal income was $40,000 to $59,999 (22.1 %) as compared to the median income of the New Zealand population in 2012 which was $29,000 [30].

Nearly all participants (97 %) had taken the antidepressants when prescribed them, and 69 % were still taking antidepressants. Just over half (51.7 %) had taken them for more than three years, and 7.8 % for less than three months. Of the 1,715 (93.8 %) who answered a question about which antidepressant they had been prescribed, the most common was Fluoxetine (22.4 %), followed by Citalopram (20.3 %), Paroxetine (8.7 %), Tricyclics (4.5 %) and Venlafaxine (2.2 %). Thirty nine percent reported that they had been prescribed multiple antidepressants.

The question analysed in this article was only completed by those who reported that they had actually taken antidepressants following a prescription (1747 participants or 97 % of the total survey sample of 1829).

### Data analysis

Participants’ responses, ranged from one word answers through to about 400 words. In order to analyse this large number of responses an initial content analysis was used to generate a quantitative description of the data and provide an overarching framework for a more detailed analysis [32]. Three pre-determined categories were used to code each participant’s response as a whole: ‘positive’, ‘negative’ and ‘mixed’ as well as a separate category for those who did not answer the question as it was intended. The coding was performed by the first author. Ten percent of responses were blind coded by the second author to check for consistency and areas of ambiguity or difference were addressed in the final coding. As there was high agreement in the initial cross-coding, further checks were not required.

Thematic analytic methods were used to identify themes within each of the three content categories. Braun & Clarke’s method provided the framework for this analysis, which involved initially identifying and coding key ideas [33]. This process was continued until the point of saturation [34]. Codes were then linked together into themes that reflected a shared meaning.

## Results

The content analysis showed that 54 % (939) of participants gave a positive account of antidepressants in their lives. Sixteen percent (273) of participants reported predominantly negative experiences and 28 % (489) described ‘mixed’ experiences. Two percent of responses did not fall into any of these categories (e.g. an elaboration of symptoms of depression).

### Positive experiences of antidepressants

In keeping with neurochemical explanations of depression many participants described antidepressants as a necessary treatment for a ‘mental illness’ often referring to the ‘serotonin deficiency hypothesis’ which sees depression as a result of a chemical imbalance, as the following participant explained: [Its] just like diabetes – a chemical shortage…I need serotonin uptake inhibitors – simple!” These participants saw antidepressants as a necessary and ongoing treatment for an underlying disease: “I would hope that one day I could stop taking them but realize that for me it is the same as taking heart pill for someone else.”

This illness framework sometimes involved resignation that the participant needed to remain on antidepressants indefinitely, as one participant explained: “My GP said that if I had diabetes I would need to take insulin forever, so not to worry that I appear to need to continue to take anti-depressants forever.”

While some participants described antidepressants as a rational treatment for a disease, other responses conveyed a stronger emotional investment in antidepressant use. A number simply described them as ‘a life-saver’ conveying a sense of the distress which had led them to take antidepressants and the relief they experienced once on antidepressants: “I can still remember the desperation and pain and if it meant taking them forever I would not hesitate.” A number of people elaborated the idea of antidepressants as a life-saver in more literal form, implying that medication had prevented them from committing suicide. As one participant put it, ‘I truly feel that I would not be alive if I had not taken them”.

A third theme within the positive responses characterized antidepressant use as enabling ‘normal’ social functioning. [Antidepressants are] the sole reason I can now function as normally as possible as a human being and a participating member of my family and community.” Part of this seemed to be that antidepressants allowed them to better fulfill the demands of their social roles. As one participant put it: “[they have been] very helpful, they have allowed me to be a better parent than I would have otherwise been, I believe.”

A fourth theme was somewhat different, describing antidepressants as a temporary way of dealing with challenging circumstances – including interpersonal and social problems. Participants alluded to a range of difficult circumstances as the following response suggestions: “[Antidepressants are] helpful in enabling me to manage the stresses of job loss and unemployment. I feel that I can cope better with job interviews on them.”

In some cases, antidepressants were seen not so much as a solution on their own but as a ‘stepping stone’ to some other kind of strategy or support as the following response suggests:

I have had such good therapy that I have been able to address the wider issues that had contributed to my mental state. …Without the medication though, I would never have had the ability to do this.

For participants who understood antidepressants this way, the medication was generally seen as a temporary solution with therapy providing the more lasting benefit.

### Negative experiences of antidepressants

For a number of participants negative experiences of antidepressants were underpinned by a belief that antidepressants were simply ineffective: “They were a waste of time and did not help me”. Some actively contested the idea that medication could be more helpful than other self-directed strategies of coping: “I get more benefit from mild to moderate exercise, or energy drinks, or spending quality time with friends.” Other participants wrote that they had initially had expectations that antidepressants would help them and had become increasingly disillusioned with their experience, as the following participant describes: “[They were] greatly disappointing. I wish I had never tried them, because before I tried them at least there was hope that something could have helped.”

The unpleasant side effects of antidepressants formed a second dominant theme in the negative category of responses with a number of participants explaining that they had struggled with the various side effects of different medicines over what appeared to be a lengthy period of time:

Each one has had a worse effect than the previous…. I can’t remember them all. It started with memory loss then progressed to me becoming borderline catatonic staring at the wall for hours unable to stand up. Within a few weeks and genuinely terrified. It was a relief to go back to the misery of depression after these experiences.

For some participants the journey to find an antidepressant that did not produce adverse effects had become the primary focus of their lives: “[It’s been] a never ending struggle to find the right ones that do not produce adverse health problems.”

Another of the prevalent theme amongst the negative experience responses related to concerns with emotional numbing and the loss of emotional authenticity. A number of participants wrote of feeling “like a zombie.” One participant elaborated on how he understood antidepressants to ‘work’: “They don’t make the problems go away. They just make me numb enough to not give a shit.” Another explained how the emotional numbness impacted on her life more generally: “By taking the medication I felt alienated from others almost as though I was walking around like a zombie in a kind of bubble.”

A related theme suggested that antidepressant use was felt to invalidate the genuine suffering participants had experienced. As the following participant put it: “In my life antidepressants have been prescribed to me to cover up what was wrong, and to me were a fake fix.” Some people also wrote about how antidepressants had led them to tolerate circumstances which they would have done better to address directly: “I believe that I stayed in a relationship that was unhealthy for me, because the antidepressants made me tolerate treatment that was unacceptable.”

A final negative theme captured the way that participants felt that antidepressants had undermined their control over their lives. For some taking antidepressants seemed to raise fears of personal weakness. For example, several participants wrote about using antidepressants as a sign of failing to “cope” or as a sign of dependency:

[It’s] like smoking. When you smoke you know it’s bad for you, but you also feel momentary relief and therefore can’t (or don’t want to stop) because you miss that feeling of being slightly more capable to handle situations.

A small number of participants linked their lack of control while on antidepressants to the lack of power over treatment options they experienced in relation to their treatment more generally:

I felt bullied into keeping taking them and at times told I would not receive therapeutic treatment if I didn’t take them. There felt like no alternative and I felt very trapped into taking them.

### Mixed experiences of antidepressants

Many participants wrote about how using antidepressants entailed a constant struggle to balance perceived benefits of the medication with side effects. For example, one participant described antidepressants as “a necessary evil, with very unfortunate side effects in terms of weight gain and sexual dysfunction which lead to me stopping the treatment despite its benefits for my mood.” Participants wrote particularly about sexual side effects and how they had to weigh up the negative impact of antidepressants on their intimate relationships with the benefits they felt antidepressants provided to these same relationships. While some, like the previous participant, had ended up stopping antidepressants because of side effects, others resigned themselves to living with these:

I know they do me good and I am better on them, but they do make me feel physically sick, and not like myself. I seem to be constantly trying life without them, but always go back to them in the end.

A second theme illustrated how participants felt grateful that antidepressants took the edge off their distress but also struggled with a sense that they did not feel ‘like themselves’:

Antidepressants have been a two edged sword. I felt less affected by things that would normally distress me while on anti-depressants… [but] when I came off them, my head felt clear, I felt like I was waking up and that I was in touch with myself again.

Another participant reflected on a similar dilemma in which she appeared to weigh up the benefits in terms of reduced feelings of depression and changes in the way she related to others:

[Antidepressants were] helpful in making my depression less. However, the effects that they had on me as a person and how I treated others is the main reason I came off them. I am a considerate and selfless person and while on the antidepressants I was the complete opposite.

A third mixed theme showed how participants actively balanced the concerns about being ‘dependent’ on them with their fears about their depression returning if they stopped taking the medication. One participant explained how she become so used to being on antidepressants and was afraid of how things would be without them:

The thing is that I have been on them so long that I have no idea what it would be like not to be on them. I would love to come off them but they have become such a ‘normal’ part of my life since I was approximately 15 years old that I am not sure I would cope without them.

Some participants implied that there had been little follow-up or advice on how to come off antidepressants as one participant wrote:

They helped me get back on my feet when I was facing a difficult time. However I was never told when to go off them and …have not heard from the doctor who prescribed them to me in years.

Participants also described how withdrawal effects made it especially difficult to come of the medication:

The withdrawal effects if I forget to take my pill are severe shakes, suicidal thoughts, a feeling of too much caffeine in my brain, electric shocks, hallucinations, insane mood swings. [I’m] kinda stuck on them now coz I’m too scared to come off it.

In contrast to responses which emphasized balancing concerns and perceived benefits of antidepressants, some attributed their mixed experience of antidepressants to the variable effects of the different medications they had been prescribed while for others they felt there had been changes in their own response to a single antidepressant over time. A few participants described how antidepressants appeared to have been initially effective but became less so over time in spite of increased dosages, and for others their response varied markedly with the different medications they had tried:

I have been on MANY different antidepressants. None of them were helpful at all to me until I tried Fluoxetine 4 years ago. My life now is greatly improved by taking this medication and a quality of life has returned.

The responses of many of these participants seemed to capture the struggle of taking different medications over time and struggling to find the one that worked for them:

I have tried almost all antidepressants available under prescription (including combinations), and most worked to varying amounts to start with, then stopped helping, then the dose was increased, then stopped working/made me worse, then dose increased to the maximum, then stopped working, then I was put on something else. I’ve wondered if I would have been better off never starting taking them at all (see Table 1 here).

**Table 1:** Content categories and themes

Content categories					
*N* 1747					
Positive experiences of antidepressants		Negative experiences of antidepressants		Mixed experiences of antidepressants	
54 % (*n* 939)		16 % (*n* 273)		28 % (*n* 489)	
Positive themes	Example of coded data	Negative themes	Example of coded data	Mixed themes	Example of coded data
Necessary for disease treatment	*No different to a diabetic taking their insulin*.	Ineffective	*Useless despite trying several different kinds*.	Benefits vs side effects	*Very unfortunate side effects in terms of weight gain and sexual dysfunction which lead to me stopping the treatment despite its benefits for my mood and anger issues*.
A life saver	*Antidepressants have been a lifeline*, *without them I would be dead.*	Unbearable side effects	*A major cost to my sex life*	Calmer but not myself	*Good at removing my anxiety and fear but it made me feel dead inside.*
Meeting social obligations	*The medication I’m on is assisting me to function as an individual and to work and contribute to the community and society and to cope with things in my workplace.*	Loss of authenticity/Emotional numbing	*Feel alienated from myself and my emotions.*	Fear of dependence versus stopping medication	*Very useful but I am now too scared to come off them and constantly worry about long term effects of being on citalopram 20mg per day*
Getting through difficult times	*Helpful for getting through a busy, tiring and stressful time in my life.*	Masks real problems	*A distraction that means I don’t address the real issue.*	Finding one that works	*Useless until I found the one that worked for me.*
A stepping stone to further help	*Provided the ‘lift’ I’ve needed to get started with other things like CBT, regular exercise etc.*	Loss of control	*A sign of failing to cope.*		

Content category: Other 2 % (*n* 46)

## Discussion

This research points to the inadequacy of asking the simple question: ‘Do antidepressants work?’ Instead, the value or otherwise of antidepressants needs to be understood in the context of the diversity of experience and the particular meaning they hold in people’s lives. Our research suggests that meanings underpinning positive experiences of antidepressants are much less homogeneous than we might have anticipated.

In spite of limited scientific support for the idea that antidepressants correct a chemical imbalance, participants have clearly been influenced by myths about ‘serotonin deficiency’ which are widely promoted to the general public [35]. However there were a range of other meanings attributed to antidepressant use which went well beyond bio-medical considerations. Some participants saw them as having both real and metaphorical life-saving properties while other suggested more temporary and pragmatic uses in relation to meeting social obligations, dealing with difficult circumstances or as a stepping stone to other forms of help. The diversity of meaning suggests that users may be appropriating the medication in different ways according to their own priorities and concerns.

This research also suggests that a large number of people may be to some extent dissatisfied with their antidepressant use. The number of participants who reported some degree of negative experience constitutes a significant proportion (44 %) of the overall sample. Some experienced little benefit from antidepressants while the side effects, particularly more subtle psychological effects such as feeling numb or ‘not like themselves,’ seemed a significant issue, which was also found to affect many participants in the analysis of the general survey data [36]. The research also raised concerns that, in some cases, managing the side effects of antidepressants might take priority over managing the depression or circumstances that helped to produce it. Participants were also concerned about the medication undermining the legitimacy of their suffering and undermining their sense of control.

Overall, the purely negative responses were far fewer than the ambivalent responses which point to the struggle that a significant proportion of people may experience in choosing to use antidepressants. The search for the right antidepressant at the right dosage may be a long and frustrating journey for some. Experimentation with dosage may also by risky insofar as research suggests that higher dosages do not necessarily produce increased efficacy but may result in greater problems with withdrawal [37]. For users, staying on antidepressants may involve an on-going negotiation between perceived benefits and the problems they are seen to cause. This research also raises concerns about whether people remain on antidepressants despite their misgivings because of fears that they would not be able to cope without medication, withdrawal effects and lack of support from their mental health providers to manage this process.

## Conclusion

It is important for mental health professionals to recognize that antidepressants are not a ‘one size fits all’ solution. They need to enter into dialogue with antidepressant users to explore the meaning antidepressants hold in their lives and the extent to which these enable or constrain their ability to make informed choices about their use. In making decisions about whether to take antidepressants, users should not be given misleading information about a known chemical aetiology in depression. They should instead be fully informed about the existing research on the efficacy of antidepressants relative to or in combination with psychosocial treatments [38]. In addition they might be usefully referred to research on user’s experiences [18] as well as receive full information about common side effects [27, 36] and withdrawal effects [28] so that they can make informed treatment choices.

The findings of this study should be interpreted in the context of its limitations. The volunteer sample might well favor responses from people who have a stronger investment in the issue, such as those who had been on antidepressants for longer. Certainly, more than half the participants in this study had been on antidepressants for more than 3 years. A volunteer sample is also likely to attract those with stronger views about antidepressants. In this study, 84 % of participants in this study answered in the affirmative to a question about whether they felt antidepressants had reduced their depression, a number well above that suggested by efficacy studies [5, 26]. This suggests that the findings of this study might over-represent positive responses to antidepressants. In addition the format of internet survey might have restricted the extent to which participants felt able to elaborate more complex or ambivalent responses.

Using an internet survey might also have skewed the sample towards a more educated and financially well-resourced group of participants and favoured a more youthful sample. The overrepresentation of women (76.6 %) is a less significant bias as they are prescribed antidepressants at much higher rates than men [39]. Concerns have been raised about limited access of potential participants to the internet but it has been recognised that this is changing quickly over time and varies considerably from one country to another [40]. In New Zealand, 80 % of households are reported to have access to internet [41].

### Ethics approval and consent to participate

This study was approved by the University of Auckland Human Participants Committee Reference Number 7340. Potential participants were directed to an online information sheet which provided details about the project including its purpose, safeguards for their anonymity and the uses to which the data would be put. They were asked to demonstrate their consent to participate by then clicking through to the survey.

### Consent for publication

Not applicable.

### Availability of data and materials

Aggregated survey data can be requested directly from the authors.
